# Prevalence and antibiotic resistance of *Staphylococcus aureus* and *Escherichia coli* isolated from raw milk in East Java, Indonesia

**DOI:** 10.14202/vetworld.2022.2021-2028

**Published:** 2022-08-23

**Authors:** Wiwiek Tyasningsih, Sancaka Chasyer Ramandinianto, Ribby Ansharieta, Adiana Mutamsari Witaningrum, Dian Ayu Permatasari, Dhandy Koesoemo Wardhana, Mustofa Helmi Effendi, Emmanuel Nnabuike Ugbo

**Affiliations:** 1 Division of Veterinary Microbiology, Faculty of Veterinary Medicine, Universitas Airlangga, Surabaya, Indonesia; 2 Department of Veterinary Public Health, Faculty of Veterinary Medicine, Universitas Airlangga, Surabaya, Indonesia; 3 Division of Veterinary Public Health, Faculty of Veterinary Medicine, Universitas Airlangga, Surabaya, Indonesia; 4 Department of Applied Microbiology, Faculty of Science, Ebonyi State University, Abakaliki, Nigeria

**Keywords:** *Escherichia coli*, extended-spectrum beta-lactamase, methicillin-resistant *Staphylococcus aureus*, public health, raw milk, *Staphylococcus aureus*

## Abstract

**Background and Aim:**

Raw milk can be a source of food-borne disease transmission and a medium for spreading antibiotic-resistant bacteria. *Staphylococcus aureus* and *Escherichia coli* are bacteria that have the pathogenic ability to attack host cells and are capable of harboring antibiotic-resistant genes. This study estimated the prevalence and antibiotic resistance of *S. aureus* and *E. coli* isolated from raw milk in East Java, Indonesia.

**Materials and Methods:**

Two hundred and fifty raw milk samples were collected from five dairy farms in East Java. *S. aureus* and *E. coli* were isolated using their respective selective media, whereas antibiotic susceptibility testing was performed using the Kirby–Bauer disk diffusion method. The methicillin-resistant *S. aureus* (MRSA) was confirmed using the oxacillin resistance screen agar test, and extended-spectrum beta-lactamase (ESBL)-producing *E. coli* was determined using the double-disk synergy test. The presence of *mec*A and *bla*TEM genes were screened by the polymerase chain reaction method.

**Results:**

Results indicated that the prevalence of *S. aureus* was 138 (55.2%) and that *E. coli* was 176 (70.4%). Of the 138 *S. aureus* isolated, 27 (19.6%) were MRSA, and among the 176 *E. coli* isolates identified, 3 (1.7%) were ESBL producers. The *mec*A gene was observed in 2 (7.4%) MRSA and all 3 (100%) ESBL-producing *E. coli* isolated harbored *bla*TEM genes.

**Conclusion:**

The presence of MRSA and ESBL-producing *E. coli* in raw milk is a serious public health threat, and public awareness should be raised about the dangers posed by these pathogenic organisms.

## Introduction

Milk is an excellent medium for bacterial growth and can be a means for spreading bacteria harmful to human health. Besides the benefits and all the nutritional values contained in it, the possibility of using milk as a medium for transmitting disease infections is quite common and often occurs in cases [1, 2]. Microorganism contamination can be found in milk if the handling does not consider hygiene aspects [3]. Efforts to fulfill the availability of milk must be accompanied by enhancing the quality and safety of dairy products because no matter how high the nutritional value of a food ingredient is, it will be useless if the food is harmful to human health [4]. Diseases transmitted from animals to humans through food are generally caused by bacterial contamination. Bacterial contamination in milk can come from poor cage management, maintenance, and unhygienic milking processes. Poor milking can cause milk to be contaminated with environmental microorganisms; thus, milk quality reduces [5]. The process of microbial contamination in milk begins when dairy cattle milk is milked; bacteria in the environment and around the udder can be carried away during the milking process if good sanitation and hygiene practices are not performed. Other contaminating milk sources include cow skins, udders, water, soil, dust, humans, and milking equipment [3].

In dairy farming in East Java, the lack of production quantity is also offset by the potential for low quality, where the feeding system, milking management, high temperature, and humidity contribute significantly to the contamination of pathogenic bacteria, such as *Staphylococcus aureus* and *Escherichia coli* [6, 7]. In line with this, Kupradit *et al*. [8] reported that in milking management, the teats of cows or the Milker’s hands have a significant effect on bacterial milk contamination. Such contamination can also occur with the movement through the intermediaries of workers, water, and production equipment [9, 10].

The milk-borne disease is a fundamental problem in the public health sector. It does affect not only human health but also the economic sector [11]. Cases of the food-borne disease have been found due to raw milk consumption [8], contamination with *S. aureus*, and *E. coli* bacteria that can come from raw milk. Thus, this study aimed to estimate the prevalence of *S*. *aureus* and *E. coli* from raw milk and the presence of crucial antimicrobial-resistant gene encodings such as the *mec*A gene in *S. aureus* and the *bla*TEM gene in *E. coli* are expected to provide a clear picture of the findings of the distribution of antimicrobial resistance (AMR) isolated from raw milk in East Java Province, Indonesia.

## Materials and Methods

### Ethical approval

Raw milk was used in this study; hence, ethical approval was not necessary. Raw milk samples were collected from five dairy farms in East Java Province, Indonesia.

### Study period and location

The study was conducted from December 2019 to March 2020. Samples were collected from 5 dairy farms in East Java Province;, Kertajaya Farm, Argopuro Farm, Suka Makmur Farm, Harapan Jaya Farm, and Semen Farm. Samples were processed at the Laboratory of the Department of Veterinary Public Health, Faculty of Veterinary Medicine, Universitas Airlangga.

### Sampling

Two hundred and fifty milk samples (25 mL each of raw milk) were obtained and 50 raw milk each from five dairy farms in East Java [12]. The samples were collected in a sterile screw-capped bottle and transported to the laboratory in an icebox within 2 h and analyzed.

### Isolation and identification of *S. aureus* and *E. coli*

The isolation of *S. aureus* and *E. coli* was done through enrichment in buffered peptone water (pH 7.0) and cultured in mannitol salt agar (Merck, Germany) and eosin methylene blue media (Merck), respectively [13, 14]. Distinct colonies of *S. aureus* were found and verified using Gram staining, catalase, and coagulase test. Distinct colonies of *E. coli* were identified and verified by growth on triple sugar iron agar and lysine iron agar, fermentative glucose degradation, citrate usage, urease production, indole fermentation, tryptophan degradation, glucose degradation, and motility.

### Antibiotic susceptibility testing of isolates

The isolates of *S. aureus* and *E. coli* were subjected to antibiotic susceptibility testing using the Kirby–Bauer disk diffusion technique as per the recommendation of the Clinical and Laboratory Standards Institute (CLSI) [15]. Briefly, Mueller-Hinton agar (Merck) was prepared according to the manufacturer’s instructions and allowed to cool to 45–50°C before pouring into plates. After the agar had solidified, plates were allowed to dry before use. An 18–24-h-old broth culture of *S. aureus* and *E. coli* isolates was standardized by diluting to 0.5 McFarland’s standard. A sterile swab stick was inserted into the standardized *S. aureus* and *E. coli* inoculum, drained to eliminate excess inoculum load, and inoculated by spreading on the surface of prepared Mueller-Hinton agar plates. After this, the inoculated Mueller-Hinton agar (Merck) plate was allowed to dry for a few minutes at room temperature (29°C) with the lid closed. After the agar surface has dried for a few minutes, antibiotic-impregnated disks of known concentrations (Oxoid, UK), oxacillin (30 μg), cefoxitin (30 μg), tetracycline (30 μg), erythromycin (15 μg), and gentamicin (10 μg) for *S. aureus*, and tetracycline (30 μg), streptomycin (10 μg), chloramphenicol (30 μg), trimethoprim (5 μg), and aztreonam (30 μg) for *E. coli*, were carefully applied on the inoculated Mueller-Hinton agar (Merck) plates using sterile forceps. The plates were then incubated at 37°C for 18–24 h, and the diameters of the inhibition zones were measured using a ruler to the nearest millimeter. Results were recorded and interpreted according to the CLSI [15].

### Confirmation test for methicillin-resistant *S. aureus* (MRSA), DNA extraction, and mecA gene detection

*S. aureus* isolates were tested for MRSA using oxacillin resistance screen agar (ORSA) (Merck) [16]. ORSA was inoculated directly with an isolated colony of *S. aureus* prepared as a liquid suspension approximately equivalent to 0.5 McFarland turbidity standards. The medium was prepared according to the manufacturer’s instructions before inoculation. The inoculated plates were incubated for 18–24 h at 37°C. The colonies showing blue indicators were recorded as MRSA, and colonies with white on the agar were recorded as methicillin-susceptible *S. aureus* after 24 h of incubation. All the MRSA verified by the ORSA were tested using a polymerase chain reaction (PCR) to detect the presence of the *mecA* gene [17]. The DNA extraction process was performed according to the QIAamp DNA Mini Kit (Promega, USA) protocol (51304 and 51306) [17]. The PCR method and primers were used as described by Ramandinianto *et al*. [18], as shown in Table-1 [18]. Positive control was *S. aureu*s ATCC BAA 1026, and negative control was *S. aureu*s ATCC 25923.

**Table-1:** Details of primers used in this study.

Primers	Sequences (5’ to 3’)	Target gene	Amplicons size	Reference
mecA-F	GAA ATG GAA CGT CCG ATA A	*mec*A	310 bp	[18]
mecA-R	CCA ATT CCA CAT TGT TTC CTA A			
TEM-F	ATA AAA TTC TTG AAG ACG AAA	*bla*_TEM_	1086 bp	[20, 21]
TEM-R	GAC AGT TAC CAA TGC TTA ATC			

### Confirmation test for extended-spectrum beta-lactamase (ESBL)-producing *E. coli*, DNA extraction, and *bla*TEM gene detection

*E. coli* isolates were studied for the presence of ESBL using the double-disk synergy test (DDST). The antibiotic disks used for DDST were amoxicillin-clavulanate (20/10 μg), cefotaxime (30 μg), and ceftazidime (30 μg) [19]. The ESBL-producing *E. coli* detected was further examined at a molecular level. Bacterial DNA was extracted using the QIAamp DNA Mini Kit (Promega) protocol according to Kristianingtyas *et al*. [19], and the *bla*TEM gene was detected using the PCR method as described by Putra *et al*. [20] and Ansharieta *et al*. [21] as indicated in Table-1. After the amplification, products were visualized by exposure of the gel to ultraviolet light and subsequently photographed and documented using agel documentation system (Promega). Positive control was *E. coli* ATCC 35218, and negative control was *E. coli* ATCC 25922.

## Results

### Prevalence and antibiotic resistance of *S. aureus*

This study indicated that of 250 milk samples taken from five dairy farms in East Java, Indonesia, 138 (55.2%) were positive for *S. aureus* isolates (Table-2).

**Table-2:** Prevalence and antimicrobial resistance profile of *S. aureus* collected from raw milk in East Java.

Location	Sample size	Confirmed *S. aureus*	Resistant to					ORSA test	*mec*A gene

			TE	OX	FOX	E	CN		
Kertajaya Farm	50	20	14	6	4	0	2	6	0
Argopuro Farm	50	30	25	10	8	5	0	8	0
Suka Makmur Farm	50	24	12	6	3	1	0	1	0
Harapan Jaya Farm	50	38	11	8	6	3	1	6	2
Semen Farm	50	26	8	8	1	6	0	6	0
Total	250	138	70	38	22	15	3	27	2
Percentage (%)	100	138/250 (55.2)	50.7	27.5	15.9	10.9	2.2	27/138 (19.6)	2/27 (7.4)

TE=Tetracycline (30 mg), FOX=Cefoxitin (30 mg), OX=Oxacillin (30 mg), E=Erythromycin (15 mg), CN=Gentamicin

(10 mg), ORSA=Oxacillin resistance screen agar test, *S. aureus*=*Staphylococcus aureus*

**Figure-1:**
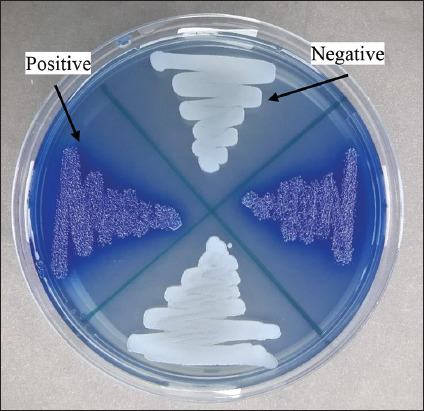
Results of oxacillin resistance screen agar test on methicillin-resistant *Staphylococcus aureus* (MRSA) isolates. Note: Positive results of MRSA are indicated by a blue indicator (aniline blue), while negative results are indicated by white/pale color indicators.

**Figure-2:**
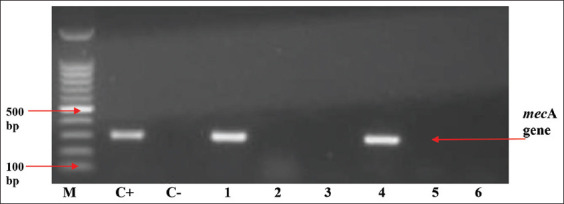
*mecA* gene on polymerase chain reaction results with positive bands at 310 bp from Harapan Jaya Farm. M line: 100 bp molecular weight markers, line C+: methicillin-resistant *Staphylococcus aureus* ATCC BAA 1026 (positive control), line C-: *Staphylococcus aureus* ATCC 25923 (negative control), lines 1 and 4: Positive isolate for *mecA* from Harapan Jaya Farm, and lines 2, 3, 5, and 6: Negative isolate for *mec*A gene.

The results of the antibiotic sensitivity test of *S. aureus* isolates in Table-2 show that different *S. aureus* isolates were found to be resistant to all the antibiotics tested. One hundred and thirty-eight *S. aureus* isolates were detected; 38 (27.5%) *S. aureus* isolates were oxacillin resistant, whereas 22 (15.9%) *S. aureus* isolates were cefoxitin resistant. In the test of tetracycline, 70 (50.7%) *S. aureus* isolates were resistant, 15 (10.9%) isolates of *S. aureus* were erythromycin resistant, and only 3 (2.2%) were gentamicin resistant. The phenotypic MRSA confirmation test was continued using the ORSA test with a blue culture indicator indicating positive confirmation results. By contrast, the white results were negative confirmation results (Figure-1). ORSA test indicated that 27 (19.6%) *S. aureus* isolates were positively confirmed MRSA, as shown in Table-2. Isolates verified as MRSA phenotypically using the ORSA method were further tested genotypically using the PCR method to detect the presence of the *mec*A gene in the isolates. Twenty-seven MRSA isolates verified by ORSA were tested using the PCR method, and two isolates (7.4% of the tested isolates) were detected to harbor the *mec*A gene (Figure-2).

### Prevalence and antibiotic resistance of *E. coli*

Of the 250 raw milk samples collected from different dairy farms, 176 samples (70.4%) were positive for *E. coli*. *E. coli* isolates were found to exhibit resistance to antibiotics such as tetracycline 30 (17.05%), streptomycin 25 (14.2%), trimethoprim 17 (9.7%), chloramphenicol 14 (7.9%), and aztreonam 3 (1.7%) isolates. The AMR profiles of the bacterial isolates are summarized in Table-3.

**Table-3:** Prevalence and antimicrobial resistance profile of* E. coli* collected from raw milk in East Java.

Location	Sample size	Confirmed *E. coli*	Resistant to					DDST	blaTEM gene

			TE	S	W	C	ATM		
Kertajaya Farm	50	35	7	5	1	4	0	0	0
Argopuro Farm	50	36	3	2	3	2	0	0	0
Suka Makmur Farm	50	30	7	5	8	1	1	1	1
Harapan Jaya Farm	50	37	9	5	1	1	0	0	0
Semen Farm	50	38	4	8	4	6	2	2	2
Total	250	176	30	25	17	14	3	3	3
Percentage (%)	100	176/250 (70.4)	17.0	14.2	9.7	7.9	1.7	3/176 (1.7)	3/3 (100)

TE=Tetracycline, S=Streptomycin, W=Trimethoprim, C=Chloramphenicol, ATM=Aztreonam, DDST=Double disk synergy test, *E. coli*=*Escherichia coli*

**Figure-3:**
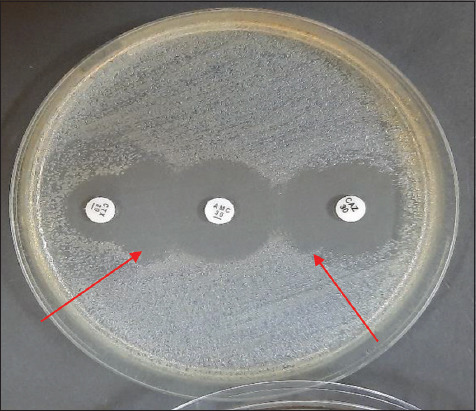
Extended-spectrum beta-lactamase-producing *E. coli* by double-disk synergy test (DDST)-positive result (red arrows showed positive synergy or keyhole effect). Note: Antibiotics disks used for DDST were amoxicillin-clavulanate (20/10 μg), cefotaxime (30 μg), and ceftazidime (30 μg).

**Figure-4:**
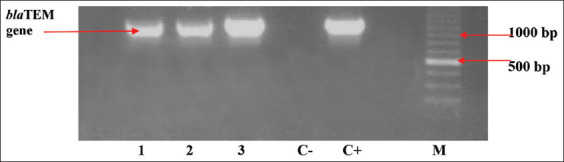
*bla*TEM gene on polymerase chain reaction results with positive bands at 1086 bp. Lane 1: Suka Makmur Farm, 2: Semen Farm, 3: Semen Farm, C-: Negative control (ATCC 25922), C+: Positive control for *bla*TEM gene (ATCC 35218), and M: Marker.

Three (1.7%) ESBL-producing *E. coli* isolates were found among 176 (70.4%) *E. coli* isolated from raw milk, and the “keyhole” effect in DDST testing is shown in Figure-3. The three isolates were tested using the PCR method to discover the encoded ESBL gene. The three positive ESBL-producing *E. coli* was observed to harbor the *bla*TEM gene (Figure-4).

## Discussion

In this study, 250 samples of raw milk were assessed; 138 (55.2%) were contaminated by *S. aureus* and 176 (70.4%) by *E. coli* isolates. The presence of bacterial contaminants in raw milk as found in this study is almost similar to a study in North India, which stated that differences could influence the differences in the number of isolates found in the study design, such as population and geographic distribution of samples, types of antibiotics used, and infection control practices [22–24]. The high level of *S. aureus* contamination of raw milk found conforms to the observation of Swetha *et al*. [25], who isolated 57.0% of staphylococci strains, of which 73.6% were *S. aureus* in dairy farms that have low milking hygiene.

In this study, *S. aureus* and *E. coli* recorded the highest antibiotic resistance to tetracycline (50.7% and 17.05%), respectively. Tetracyclines have the highest antibiotic resistance because they are often used in veterinary medicine, and other antibiotics used in this study such as beta-lactams such as oxacillin (27.5%) and cefoxitin (15.9%), macrolides such as erythromycin (10.9%), and aminoglycosides such as gentamicin (2.2%). The use of broad-spectrum antibiotics such as tetracyclines and beta-lactams is more common in cases of clinical mastitis in dairy cattle because of their effective treatment results. Twenty-seven (19.6%) MRSA isolates were validated using ORSA, and the highest percentage was detected in Argopuro farms, as indicated in Table-2. The presence and detection of MRSA in raw milk, as observed using the ORSA test, is in agreement with the study by Ramandinianto *et al*. [18] and Yunita *et al*. [26], where the presence of MRSA was observed by the ORSA test. It was also deduced that the blue culture indicator showed positive confirmed results, whereas white is negative confirmed results [26].

Handling unclean and unhygienic food during the production process, packaging, and distribution plays an important role in food poisoning [27]. Other researchers have stated that cow milk can transmit different pathogens, including strains of staphylococci [28]. Research on antimicrobial drug resistance of *S. aureus* reports that dairy product-related contamination is widespread globally. Some researchers report that bacterial outbreaks in milk and dairy products in countries are approximately 2–6% [29]. MRSA is resistant to all beta-lactam antibiotics, including cephalosporins and monobactams, an essential group of antibiotics for treating staphylococcal infections [22] and agreed with the results of this study. MRSA infection causes therapeutic problems and facilitates its spread, necessitating rapid and early diagnosis and accurate MRSA identification [30]. In this study, of *S. aureus* isolates, 27.5% were found to be resistant to oxacillin and 15.9% to cefoxitin in the disk diffusion method.

Presumptive MRSA can be made using oxacillin and cefoxitin. Brown and Walpole [31] stated that MRSA detection using phenotypic methods still does not indicate optimal results, and *mec*A genotype testing remains the major recommendation even though it cannot be applied to routine testing. To identify accurate MRSA, fast and cost-effective, a phenotypic technique with the ORSA test can be used [32]. Cefoxitin and oxacillin disk diffusion have the same sensitivity level of 100%. In specificity, cefoxitin disk diffusion was 92.59%, whereas oxacillin disk diffusion was 74.07% [22]. A study conducted by Boubaker *et al*. [33] indicated that the cefoxitin disk method has a better sensitivity level than the oxacillin disk technique in detecting MRSA. Therefore, the oxacillin disk technique still has a false-positive rate.

All ORSA-positive isolates were genotypically tested using PCR to detect the presence of the *mec*A gene, the gold standard for detecting MRSA. Two (7.4%) *S. aureus* isolates from the Harapan Jaya Farm were discovered to have the *mecA* gene. Cefoxitin is an excellent inducer to express the presence of the *mec*A gene because it can increase the expression of penicillin-binding protein 2a, encoded by the *mec*A gene [18]. The results of this study show that milk contamination by MRSA can be caused by different factors, one of which is low milking hygiene. MRSA contamination is hazardous to public health; it increases the potential for the spread of difficult-to-treat staphylococcal infections. It needs the ability to accurately, quickly, and cost-effectively identify MRSA contamination in transmission media such as food of animal origin. Genotypic detection using PCR to detect the presence of the *mec*A gene is the gold standard for MRSA detection; however, there are still numerous laboratories that cannot conduct molecular testing; cefoxitin diffusion can be used as a marker for MRSA detection. This is based on the cefoxitin disk diffusion test’s ability to detect the expression of the *mec*A gene so that it can be a solution as a more effective and efficient MRSA screening instrument in terms of cost and technical applications.

The results also indicated that the prevalence of *E. coli* found in milk was 70.4%. These data show the poor sanitation practices of farmers during the milking process [34]. This figure is similar to that reported by Chey *et al*. [35], stating that the prevalence of *E. coli* was highest (72.2%) in raw milk. In line with other developing countries, namely Bangladesh, as much as 75% of the milk samples studied contained *E. coli* [36]. Tetracyclines have the highest antibiotic resistance of 17.0% because they are commonly used in veterinary medicine, and other antibiotics used in this study, such as aminoglycosides such as streptomycin (14.2%), sulfonamides such as trimethoprim (9.6%), and macrolides such as chloramphenicol (7.9%). Broad-spectrum antibiotics such as tetracyclines and beta-lactams are more common in cases of clinical mastitis in dairy cattle in Indonesia because of their effective treatment results. The tetracycline and aminoglycoside groups are the first-choice antibiotics for respiratory and digestive tract problems. By contrast, the second choice is the macrolide and sulfonamide-trimethoprim drug combinations, which significantly affect rumen microbial activity. The last choice is the third- and fourth-generation antibiotics from cephalosporins. By contrast, the combination of sulfonamide-trimethoprim drugs significantly affects the rumen microbial activity, and the last resort is the third-generation cephalosporins [37]. Three ESBL-producing *E. coli* (1.7%) isolates were identified from raw milk. The discovery of ESBL *Enterobacteriaceae* (*E. coli*) originating from milk shows the presence of environmental pollution and a lack of environmental sanitation when milking is performed [38]. *E. coli* is a bacterium that can be a reservoir of different antibiotic resistance genes [39], including beta-lactam antibiotic resistance genes, which make *E. coli* capable of producing beta-lactamase enzymes [40]. ESBL enzymes are produced by many strains belonging to the *Enterobacteriaceae* family. These bacteria can hydrolyze penicillins and third-generation cephalosporins, monobactam, and other antibiotics, except for carbapenems [41]. These enzymes are mainly encoded by many specific genes, namely, the *bla*SHV, *bla*CTX-M, and *bla*TEM genes [42]. Sanitation of the cage, the bottom of the cage, and the drainage of the cage need to be considered by farmers to prevent contamination of milk by suspected ESBL-producing bacteria. The occurrence of antibiotic resistance originates from bacterial plasmids that can accommodate resistance genes and spread them to other bacteria [43]. Different resistance genes can accumulate in bacterial plasmids, usually in the R (resistance) plasmid, which is the reason for finding bacterial isolates that are resistant to different antibiotics and can create new gene sequences [44].

The prevalence of the *bla*TEM genes in ESBL-producing *E. coli* was 3 (1.7%). This finding is in line with the research conducted by Ansharieta *et al*. [21], who stated that *E. coli* contamination found in milk from dairy farms tends to encode the *bla*TEM gene in ESBL-producing *E. coli* bacteria. These results show that pathogenic *E. coli* originating from food of animal origin are also exposed to antibiotics and can transfer these genes to other pathogenic bacteria under certain conditions [45]. Therefore, the presence of ESBL bacteria in raw milk is quite dangerous. ESBL-producing *E. coli* strains obtained from raw milk samples are of particular concern because these pathogens can affect human and calf consumers and cause the spread of this antibiotic-resistant pathogen to humans and animals [46]. During lactation, ESBL-producing *E. coli* can also be found in raw milk with or without mastitis symptoms. This shows that the cleanliness of the cage that contaminates the milk cage is also a risk factor for ESBL-producing organisms, which can contaminate raw milk products [47, 48].

Therefore, genetic evidence encoding MRSA and ESBL-producing *E. coli* can be used to confirm interactions at the microbial level in humans and animals, especially between commensal and pathogenic bacteria, facultative and obligate bacteria in the same environment, and horizontal gene transfer of the bacteria making the distribution. An integrative approach such as “One Health” is needed to understand and identify the possibility of preventing the spread of MRSA and ESBL-coding genes and infection in humans [49]. The application of the concept of One Health integration is assumed to accelerate disease prevention and prediction to control these bacteria [50].

Food-borne disease is a significant concern worldwide. This is a leading problem in developing countries that lack high sanitation management during collecting and processing cow’s milk. As seen in this study, *S. aureus* and *E. coli* contamination found in raw milk can be caused by cross-contamination of milk with feces or by a lack of hygienic measures during milk collection and processing [9]. According to Ukah *et al*. [51], a factor causing antibiotic resistance in humans is consuming food of animal origin in raw or undercooked form. A multisectoral approach to medical treatment in veterinary medicine and animal food production can realize global cooperation in controlling the ecological development of antibiotic resistance for public health [52].

## Conclusion

The presence of MRSA and ESBL-producing *E. coli* in raw milk is a serious public health threat, and public awareness should be raised about the dangers posed by these pathogenic organisms. Evidence by molecular identification indicated the presence of *mec*A and *bla*TEM genes in *S. aureus* and *E. coli* found in raw milk obtained from five dairy farms in East Java, Indonesia. Although the results indicated that MRSA and ESBL-producing *E. coli* from raw milk had a relatively low prevalence at the molecular level, MRSA and ESBL-producing *E. coli* in the food chain is a potential threat if not controlled since it can spread from animals to humans.

## Authors’ Contributions

MHE and WT: Conceptualization and supervision of the study and drafted the manuscript. MHE, SCR, and RA: Data curation. WT and AMW: Formal analysis. AMW, DAP, and DKW: Investigation. MHE and AMW: Methodology. DAP, DKW, and AMW: Project administration. MHE, SCR, and RA: Resources. MHE, WT, and ENU: Validation. SCR, RA, and AMW: Visualization. MHE and ENU: Review and editing. All authors have read and approved the final manuscript.
